# A Comparative Nonrandomised Observational Study of Different Modalities of Renal Replacement Therapy (Continuous Renal Replacement Therapy vs. Sustained Low Efficiency Dialysis) With Acute Kidney Injury in an Intensive Care Unit Setup: A Pilot Study

**DOI:** 10.7759/cureus.88678

**Published:** 2025-07-24

**Authors:** Choudhury Sudhiranjan Dash, Rishit Harbada, Rushi V Deshpande, Amjad Khan Pathan

**Affiliations:** 1 Nephrology, Sir H. N. Reliance Foundation Hospital and Research Centre, Mumbai, IND; 2 Nephrology, Jaslok Hospital and Research Center, Mumbai, IND

**Keywords:** acute kidney injury, continuous renal replacement therapy (crrt), sustained low-efficiency dialysis (sled), vasopressor dependency, vasopressor index

## Abstract

Background: This study investigated the comparable efficacy of sustained low-efficiency dialysis (SLED) over continuous renal replacement therapy (CRRT) in maintaining hemodynamic stability in critically ill patients with acute kidney injury (AKI) in an intensive care unit (ICU).

Methods: A single-centre, prospective observational study was conducted at Jaslok Hospital and Research Centre, Mumbai, involving 67 patients requiring RRT in the ICU. Thirty-five patients were included in the CRRT (Baxter Prismaflex System, USA) cohort, and 32 patients were included in the SLED (Fresenius 4008S, Fresenius Medical Care, Bad Hamburg, Germany) cohort. A total of 58 sessions of CRRT and 87 sessions of SLED were analysed.

Results: The median duration of CRRT was 1.93 days, whereas the median duration of SLED was 0.73 days. The mean duration of dialysis and mean ultrafiltration rate per session were 46.48+/-37.65 hours and 17.61+/-13.2 hours, and 23.77+/-26.72ml/hr and 103.51+/-108 ml/hr, respectively, in the CRRT and SLED cohorts. Metrics such as the Delta VI, Delta VD, Sequential Organ Failure Assessment (SOFA), and Acute Physiological Assessment and Chronic Health Evaluation (APACHE-2) Scores were evaluated within 48 hours of therapy initiation, along with 28-day mortality rates and complication assessments. The mortality rates at 48 hours and all-cause mortality rates at 28 days were 45.71% and 50% and 77.14% and 78.12% between the CRRT and SLED cohorts, respectively. Between survivors and nonsurvivors, the SOFA and APACHE-2 scores showed statistical significance. A SOFA score of ≥11 and an APACHE II score of >20 are associated with a negative short-term outcome. During the process of therapy, complications of clinical significance were filter clotting, hypokalemia, hypophosphatemia, and arrhythmias. Although we could not observe statistically significant differences between the groups, they could still contribute towards untoward outcomes if left untreated.

Conclusion: While both therapies showed similar baseline characteristics and outcomes, the SLED demonstrated a potential advantage in terms of reduced filter clotting incidents and logistics, making it an attractive alternative in resource-limited settings. The results highlight the equivalent efficacy of the SLED in delivering RRT while supporting hemodynamic stability in critically ill patients.

## Introduction

Acute kidney injury (AKI) is one of the earliest clinical conditions to be described in the medical literature. William Herbenden first mentioned acute renal failure (ARF) in the early 20th century, as early as 1902, and coined the term ischuria renalis. Since then, the nomenclature for ARF has been redefined 35 times in different studies [1]. To resolve this confusion, the term ARF was replaced by AKI.

AKI is defined as a rapid decline in the glomerular filtration rate occurring over minutes to days, with retention of nitrogenous waste products, namely, blood urea nitrogen, creatinine, and others [2]. Depending on the deﬁnition used, the incidence of AKI ranges from 1% to 25% in intensive care unit (ICU) patients, with mortality rates ranging between 15% and 60% [2-4]. AKI-associated mortality has changed little over the years despite advances in medical care, mostly due to the increasing age and the complexity of patients suffering from AKI. AKI is one of the most common and serious complications in critically ill patients admitted to the ICU and is considered an independent risk factor for morbidity and mortality. The term AKI encompasses the entire spectrum of the syndrome from minor changes in markers of renal function to the requirement for renal replacement therapy (RRT) [5].

To restore the systemic milieu in critically ill patients, different modalities of RRT are offered when indicated. These include intermittent hemodialysis (IHD), prolonged intermittent renal replacement therapy (PIRRT), sustained low efficiency dialysis (SLED), and continuous renal replacement therapy (CRRT). In critically ill patients with hemodynamic instability, the latter three are more appropriate. There is a debate in the literature regarding the superiority of convective clearance of solute via CRRT vs. diffusive clearance via SLED. Convective clearance is considered less efﬁcient than diffusive clearance, but its continuous nature compensates for its drawback; moreover, bulk solute removal with CRRT is reportedly greater than that with IHD. Other advantages of CRRT over IHD include better hemodynamic stability and achievement of the ultrafiltration goal in a controlled environment. In addition, there is a better control of acid/base and electrolyte status, uremic toxins, and inflammatory mediator removal, particularly in patients with systemic inﬂammatory response syndrome (SIRS). SLED is a hybrid modality that has components taken from both CRRT and IHD, with an efficacy equivalent to that of CRRT and fewer disadvantages (cost, resources, and mobility restrictions for any procedure). These advantages of SLED have been widely reported in medical literature; however, they are not universally accepted. Despite the advancements in the understanding and management of AKI, including the introduction of RRT, mortality in ICU patients with AKI remains largely unimproved and unacceptably high [6]. Mortality in ICU patients with AKI severe enough to require RRT has been reported to be as high as 80% [7,8].

Therefore, this study aimed to compare the two modalities of RRT, i.e., SLED vs CRRT, with the assumption that SLED is not inferior to CRRT in terms of hemodynamic stability, complications, and dependence on RRT in critically ill patients with AKI. The study evaluated the hemodynamic stability of critically ill patients with AKI requiring RRT using two objective assessment tools, delta vasopressor index (DVI) and delta vasopressor dependency (DVD). Furthermore, we studied the early clinical deterioration in critically ill patients with AKI on RRT, defined as a rise in the Sequential Organ Failure Assessment (SOFA) score or death within 48 hours after starting RRT and mortality at 28 days after RRT initiation. The secondary objectives of the study included assessing the complications associated with the two RRT modalities and the persistent RRT dependence at 28 days.

## Materials and methods

Study design

This single-centre prospective observational study was carried out at Jaslok Hospital and Research Centre, Mumbai. The study was designed in accordance with the reporting guidelines endorsed by the Consolidated Standards of Reporting Trials (CONSORT) and the Strengthening of Observational Studies in Epidemiology (STROBE) statement [9]. The institutional review board approved the study after obtaining ethics committee approval (41st Ethics Committee Meeting/dated 05.08.2017), and it was performed in accordance with the Declaration of Helsinki. Informed consent was obtained from all patients or their next of kin or legal heir (for patients incapable of giving consent).

Study population

Patients with AKI requiring RRT admitted to the ICU between September 2017 and February 2019, who received SLED or CRRT, were included in the study. The inclusion criteria were age ≥18 years with AKI requiring RRT. Critically ill patients with chronic kidney disease (CKD) requiring RRT and patients with AKI who did not require RRT and were managed conservatively were excluded from the study

Sample size (n) calculation

The sample size for this study was calculated using the following formula described by Wan et al. [10]:

 $S &asymp;(q3&minus;q1) / 2&Phi; &minus;1[(0.75n&minus;0.125) / (n+0.25)]$,

where S = standard deviation; q1 = first quartile; q3 = third quartile; Φ −1 is the inverse cumulative distribution function for normal distributions; n = sample size.

Using the above formula, the final sample size was computed as n = 60, with a minimum of 30 participants in each arm. Assuming a 10% dropout rate, the final sample size was inflated to n = 66.

Methodology

In this study, 67 patients, 35 inthe CRRT cohort and 32 in the SLED cohort, who fulfilled the inclusion criteria, were studied over a period of 18 months.

A complete thorough clinical examination and blood investigations were performed by the primary treating team, intensivists, and treating nephrologists. Preliminary clinical data, including patient demographics, comorbidities, severity scores (SOFA and APACHE II), AKI stage according to the KDIGO, and the aetiology of AKI, were collected. Laboratory parameters, including serum creatinine levels, arterial blood gas analysis (ABG) levels, electrolytes, lactate levels, procalcitonin levels, routine urine analysis, as well as hemodynamics, including blood pressure and fluid balance, were also collected.

Sepsis was defined as per the 2018 Surviving Sepsis Guidelines [11]. The source of sepsis was defined as the infection focus studied and was chiefly categorised as respiratory, intra-abdominal, which also included genito-urinary infections, haematological, or other infections. The decision to initiate RRT, the timing of RRT initiation, and the modality of RRT were made by the treating nephrologist. All measurements and data were collected by the same investigator.

A Fresenius 4008S hemodialysis machine (Fresenius Medical Care, Bad Hamburg, Germany) and a 1.4 m2 dialyser membrane (Polyflux Gambro, USA) were used for the patients undergoing SLED, while a CRRT machine (Baxter Prisma Flex System, USA) with an M-100 Prismaflex dialyser and the extra-corporeal circuit was used for patients undergoing CRRT. Dialysis duration, the total number of dialysis sessions, blood flow (Qb), dialysate flow (Qd), prescription, anticoagulation during dialysis, and ultrafiltration rates were determined by the treating nephrologist. Vasopressors were used to target a mean arterial pressure (MAP) of >65 mmHg after adequate fluid resuscitation.

Hemodynamic metrics were described via two objective parameters, vasopressor index (VI) and vasopressor dependency (VD). Hemodynamic stability was calculated using the formula given by Mishra et al. [12]. The dose of vasoactive/vasopressor agent is expressed as the inotropic score (vasopressor index), a dimensionless variable calculated as: [(dopamine dose x 1)+(dobutamine dose x 1)+(adrenaline dose x 100)+(noradrenaline dose x100)+(phenylephrine dose x 100)+(vasopressin dose x 10)], where all doses are expressed as µg/kg/min, except vasopressin, which is expressed in units/h.

The modification to include vasopressin was considered as it is the second most common inotrope used in the ICU after norepinephrine. This score has also been referred to as the vasopressor score or catecholamine index [13-16]. This dose-response relationship is expressed as the vasopressor dependency index, which is calculated as the ratio of the inotropic score to MAP; the higher the score, the greater the vasopressor requirement. This step is performed to negate the effect of the MAP.

Vasopressor Index (VI) - [(dopamine dose x 1) + (Dobutamine dose x 1) + (adrenaline dose x 100) + (noradrenaline dose x100) + (phenylephrine dosex100) + (Vasopressin dose x 10)]

Vasopressor Dependency (VD) = VI/MAP x 100

The delta VI (∆VI) and delta VD (∆VD) were calculated as the difference between the predialysis value and the worst recorded value during the dialysis session. The comparison was performed by analysing the worst ∆VI and ∆VD value for each patient. In addition, other outcome parameters, such as mortality within 48 hours, change in SOFA score within 48 hours of RRT initiation, and complications during dialysis sessions, were recorded.

Objectives of the study

Primary Objectives

The primary objectives of this study are 1) to examine early clinical deterioration in critically ill patients with AKI on RRT, defined as a rise in SOFA score or death within 48 hours after starting RRT and to study mortality at 28 days after RRT initiation, and 2) to compare the different modalities of RRT utilised in critically ill patients with AKI and to determine whether prolonged intermittent renal replacement therapy (PIRRT)/slow low-efficiency extended dialysis (SLEED) is noninferior to continuous renal replacement therapy(CRRT).

Secondary Objective

The secondary objectives of this study are 1) to examine complications of RRT in both groups of patients and 2) to study persistent RRT dependence at 28 days.

Statistical analysis

Statistical analysis was performed using GNU PSPP version 0.10.1. Qualitative data are presented as frequency and percentage. The quantitative data are presented as mean ± SD. Categorical variables were compared using the chi-square or Fisher’s exact tests. A t-test was used to compare parametric variables. For nonparametric variables, the Mann-Whitney U Test was used to compare two groups. The odds ratio was calculated using the IBM SPSS Statistics for Windows, Version 25.0 (IBM Corp., Armonk, NY) software. A two-sided probability (P) value <0.05 was considered statistically significant. The results were graphically represented where deemed necessary. As the study was a non-randomised observational study to reduce sampling bias, a logistic regression analysis was carried out for the variables.

## Results

A total of 67 patients (35 in the CRRT cohort and 32 in the SLED cohort) were included in the study.

In our study, the baseline characteristics of the two comparison groups, i.e. the CRRT and SLED, were similar (Table 1). The SOFA and APACHE 2 scores did not statistically differ between the two groups. In addition, baseline laboratory and pre-dialysis variables were similar in both groups. The proportion of females in the CRRT group (23%) was less than that in the SLED group (47%), which showed a statistically significant value.

**Table 1:** Baseline characteristics of patients in the CRRT and SLED groups SOFA: Sequential Organ Failure Assesment, APACHE II: Acute Physiology and Chronic Health Evaluation, COPD: chronic obstructive pulmonary disease, HRS: hepatorenal syndrome, TLC: total leucocyte count, APTT: activated partial thromboplastin time, INR: International Normalised Ratio, CRRT: continuous renal replacement therapy, SLED: sustained low-efficiency dialysis

Variable	CRRT(N = 35)	SLED (N = 32)	Test value	P-value
Mean age (years)	56.17 ± 14.13	59.72 ± 14.08	1.03	0.31
Gender distribution				
Males: females	27:8	17:15	4.28	0.0701
Severity scoring				
SOFA score	11 ± 2.44	11.16 ± 2.61	0.30	0.766
APACHE II score	21.65 ± 8.24	24.81 ± 8.66	1.53	0.131
Comorbidities				
Diabetes mellitus	16 (50%)	14 (43.75%)	0.26	0.872
Hypertension	16 (50%)	18 (56.25%)	0.123	0.726
COPD	1 (2.94%)	3 (9.37%)	1.265	0.261
Ischemic heart disease	7 (20%)	7 (21.87%)	0.26	0.85
Aetiology of AKI				
Sepsis	20 (57.1%)	22 (68.75%)	0.963	0.326
Cardiogenic shock	7 (20%)	4 (12.5%)	0.685	0.408
Liver AKI (HRS)	7 (20%)	4 (12.5%)	0.685	0.247
Tumour lysis syndrome	1 (2.9%)	2 (6.25%)	0.450	0.502
Laboratory parameters				
Hemoglobin g/dL	9.69 ± 1.38	9.3 ± 1.54	1.208	0.23
TLC cu/ml	17089 ± 8395.3	16751 ± 8621	0.163	.871
Platelets x10^9 ^/L	143.25 ± 56.85	176.75±80.74	1.981	.052
Aptt (seconds)	35.13 ± 4.96	33.72±3.83	1.293	0.201
INR	1.72 ± 0.57	1.6 ± 0.45	0.948	0.35
Procalcitonin (ng/ml)	28.3 ± 45.8	30.4 ± 33.2	0.213	0.83
Lactate (mmol/L)	8.94 ± 4.06	10.71 ± 4.67	1.66	0.102
Pre-dialysis variables				
Creatinine (mg%)	3.31 ± 1.79	4.21 ±2.22	1.819	0.074
pH	7.26 ± 0.12	7.29 ± 0.085	0.952	0.344
Bicarbonate (mEq/L)	13.95 ± 4.88	12.26 ± 5.14	1.373	0.175
Potassium (mEq/L)	5.15 ± 1.03	5.29 ± 0.99	0.595	0.554

Table 2 shows a comparison of the indications for RRT initiation between the two groups. There were no significant differences noted between the two groups.

**Table 2:** Indication for RRT initiation RRT: renal replacement therapy, CRRT: continuous renal replacement therapy, SLED: sustained low-efficiency dialysis

Indication for RRT	CRRT	SLED
Anuria	16 (45.7%)	15 (46.88%)
Metabolic acidosis	8 (22.86%)	6 (18.75%)
Uremia/azotemia	6 (17.14%)	6 (18.75%)
Hyperkalemia	5 (14.29%)	5 (15.63%)
Total	35(100%)	32(100%)

A total of 58 sessions of CRRT were analysed. The median number of days per patient was 1.93 days. The mean duration of dialysis was 46.48 ± 37.65 hours. The mean blood flow (Qb) in these sessions was 110.29 ± 18.1 ml/min. The mean dialysate flow (Qd) was 1.064 ± 194.6 L/h. The mean ultrafiltrate removal per session was 23.77± 26.72 ml/h.

Eighty-seven sessions of SLED were analysed. The median number of days per patient was 0.73 days. The mean duration of dialysis was 17.61 ± 13.2 hours. The mean blood flow (Qb) in these sessions was 120.63 ± 19.33 ml/min. The mean dialysate flow (Qd) was 18.0 ± L/h. The mean ultrafiltrate removal rate per session was 103.51 ± 108 ml/h.

The predialysis VI was 38.35 ± 28.37 and 45.74 ± 26.39 in the CRRT and SLED cohorts, respectively. The intra-dialytic VI was 77.48 ± 45.07 and 81.5 ± 42.44 in the CRRT and SLED cohorts, respectively. The intradialytic hypotension, which was measured in terms of ΔVI, in the CRRT group (39.13 ± 24.17) was comparable to that in the SLED group (35.77 ± 26.78). The predialysis MAP was 73.22 ± 6.89 mmHg and 72.15±7.18 mmHg in the CRRT and SLED cohorts, respectively. The mean MAP recorded was 67.22 ± 6.92 mmHg in the CRRT group and 66.87 ± 5.717 mmHg in the SLED group. In our study, the hemodynamic variables remained comparable between the two cohorts (Table 3).

**Table 3:** Mean of the hemodynamic variables in the study groups VI: vasopressure index, VD: vasopressure dependence, MAP: mean arterial pressure, Delta VI: Delta VI, Delta VD: Delta VD, CRRT: continuous renal replacement therapy, SLED: sustained low-efficiency dialysis Delta VI and Delta VD were calculated as the difference between the predialysis value and the worst recorded value during the dialysis session.

Hemodynamic variables	CRRT	SLED	t-value	P-value
Vasopressor index (VI) (predialysis)	38.35 ± 28.37	45.73 ± 26.39	1.10	0.276
Vasopressor dependence (VD) (predialysis)	54.76 ± 44.97	65.84 ± 41.29	1.047	0.299
Mean arterial pressure (MAP) (predialysis)	73.22 ± 6.89	72.15 ± 7.18	0.623	0. 535
VI (intradialytic)	77.48 ± 45.07	81.5 ± 42.44	0.375	0.709
VD (intradialytic)	121 ± 81.65	125.79 ± 71.05	0.256	0.80
MAP (intradialytic)	67.22 ± 6.92	66.87 ± 5.717	0.227	0.821
Delta VI	39.13 ± 24.17	35.77 ± 26.78	0.541	0.591
Delta VD	66.24 ± 47.11	59.94 ± 47.28	0.545	0.587

The vasopressor dependency was 54.76 ± 44.97 in the CRRT cohort and 65.84 ± 41.29 in the SLED cohort. The VDs were 121 ± 81.65 and 125.79 ± 71.05 in the CRRT and SLED cohorts, respectively. There was no statistically significant difference in the intradialytic hypotension, which was measured in terms of ΔVD, between SLED (59.94 ± 47.28) and CRRT (66.24 ± 47.11) groups. Both modalities were equal in terms of hemodynamic tolerability, with no statistically significant difference. A regression analysis was carried out for ΔVI and ΔVD, respectively, between the SLED and CRRT groups (Tables 4, 5). There was no significant difference elicited between the two groups. ANOVA performed considering Delta VI and Delta VD as dependent variables did not show any statistical difference.

**Table 4:** Regression analysis on the Delta vasopressor index (ΔVI) between CRRT and SLED a. Dependent variable: VIDiff b. Predictors: (Constant), APACHEScore_PreDailysis, APTT (Activated Partial Thromboplastin Time), comorbid, CREATININE, PCT (Procalcitonine), OUTCOME, GROUP GROUP, K (Potassium), SEX, Agegr, PT_INR (International Normalise Ratio), HB (Hemoglobin), TLC (Total Leucocyte Count), LACTATE, PLATELET, SOFAscore_Predailysis, Bicarbonate, Ph CRRT: continuous renal replacement therapy, SLED: sustained low-efficiency dialysis

Model	Unstandardised B	Coefficient standard error	Standardised coefficient beta	t	sig
Constant	-203.566	447.225		-0.455	0.651
Sex	-2.830	7.991	-0.053	-0.354	0.725
Comorbid	-2.384	3.952	-0.098	-0.603	0.549
Hb (gm%)	-2.252	2.637	-0.130	-0.854	0.397
Total leucocyte count (TLC)cu/ml	0.000	0.001	0.148	0.825	0.414
Platelet x10^9 ^/L	-0.017	0.069	-0.048	-0.248	0.805
PT_INR (seconds)	-7.063	8.935	-0.144	-0.791	0.433
APTT (seconds)	0.075	0.864	0.013	0.087	0.931
PCT (ng/ml)	0.012	0.098	0.019	0.123	0.902
Lactate (mmol/l)	0.695	1.075	0.121	0.646	0.521
Creatinine (mg%)	-.3.525	1.862	-0.285	-1.893	0.064
pH	31.816	63.057	0.137	0.505	0.616
Bicarbonate (meq/l)	1.133	1.287	0.226	0.880	0.383
Potassium (meq/l)	1.843	3.867	0.074	0.477	0.636
Group-Grp	-3.526	8.856	-0.070	-0.398	0.692
Outcome	-8.850	9.218	-0.147	-0.960	0.342
Age group in years (Group 1 <50, Group 2 51-60, Group 3 61-70, Group 4 >71 )	0.047	3.995	0.002	0.012	0.991
SOFA score (predialysis)	2.182	2.189	0.216	0.997	0.324
APACHE 2 score (predialysis)	0.647	0.861	0.227	0.783	0.437

**Table 5:** Regression analysis on Delta vasopressor dependence (ΔVD) between CRRT and SLED a. Dependent variable: Delta VD b. Predictors: (Constant), APACHEScore_PreDailysis APACHE Score_PreDailysis, APTT, comorbid, CREATININE, PCT, OUTCOME, GROUP GROUP, K, SEX, Agegr, PT_INR PT_INR, HB, TLC, LACTATE, PLATELET, SOFAscore_Predailysis SOFAscore_Predailysis, HCO3, PH CRRT: continuous renal replacement therapy, SLED: sustained low-efficiency dialysis

Model	Unstandardised B	Coefficient standard error	Standardised coefficient beta	t	sig
Constant	174.362	819.292		0.213	0.832
Sex	-.7.310	14.639	-0.074	-0.499	0.620
Comorbid	-.3.351	7.241	-0.075	-0.463	0.646
Hb (gm%)	-4.183	4.832	-0.131	-0.866	0.391
TLC (cu/ml)	0.001	0.001	0.169	0.955	0.344
Platelet x10^9^/L	-0.016	0.126	-0.024	-0.128	0.898
PT_INR	-12.703	16.369	-0.140	-0.776	0.442
APTT (seconds)	0.005	1.582	0.001	0.003	0.997
PCT (ng/ml)	0.050	0.179	0.042	0.278	0.783
Lactate (mmol/l)	.718	1.969	0.068	0.365	0.717
Creatinine (mg%)	-5.562	3.410	-0.242	-1.631	0.109
pH	-16.164	115.517	-0.038	-0.140	0.889
Bicarbonate (meq/l)	2.026	2.358	0.218	0.860	0.394
Potassium( meq/l)	1.957	7.084	0.042	0.276	0.783
Group-Grp	-4.184	16.224	-0.045	-0.258	0.798
Outcome	-17.781	16.888	-0.159	-1.053	0.298
Age group in years (group 1 <50, Group 2 51-60, Group 3 61-70, Group 4 >71)	1.181	7.319	0.027	0.161	0.872
SOFA Score-Predialysis	4.153	4.011	0.222	1.035	0.306
APACHE 2 Score_Predialysis	0.971	1.578	0.176	0.616	0.541

The signs of early clinical deterioration were mortality within 48 hours of RRT initiation and an increase in the SOFA and APACHE scores. Among the 67 patients who underwent RRT, all-cause mortality at 28 days was 77.14% in the CRRT cohort and 78.12% in the SLED cohort. The mortality rates at the end of 48 hours were 45.71% and 50% in the CRRT and SLED cohorts, respectively. The mortality rate within 48 hours of initiation in the CRRT cohort was lower than that in the SLED cohort. However, there was no statistically significant difference in mortality between the two groups (Table 6). The relative risk of mortality within 48 hours was 1% higher in the SLED group, but not statistically significant (RR = 1.09 with 95% confidence interval 0.66-1.8, p = 0.72). The relative risk of mortality was equal in both groups, while for mortality at 28 days, the RR was 1.01 (95% CI, 0.78-1.3, p = 0.92).

**Table 6:** Comparison of dependence on RRT among the study subjects RRT: renal replacement therapy, CRRT: continuous renal replacement therapy, SLED: sustained low-efficiency dialysis

Variable	CRRT	SLED	P-value
Dependence on RRT	2 (40%)	3 (42.85%)	0.46
Mortality within 48 hours and within 28 days of RRT initiation			
	CRRT	SLED	P-Value
Mortality within 48 hours of initiation of RRT	16 (45.71%)	16 (50%)	0.73
Mortality within 28 days of initiation of RRT	27 (77.14%)	25 (78.12%)	0.92

The mean APACHE score among the non-survivors in the CRRT group was 22.7 ± 7.7, whereas that of the survivors was 18.12 ± 9.24. The mean APACHE-2 scores of the non-survivors and survivors of the SLED group were 25.52 ± 9.34 and 8.71 ± 2.36, respectively. The survivors in both groups had lower APACHE scores, but the difference was not statistically significant (Table 7).

**Table 7:** Mean comparison of APACHE 2 score and SOFA score between survivors and non-survivors SOFA: Sequential Organ Failure Assessment, APACHE-2: Acute Physiological Assessment and Chronic Health Evaluation, SLED: sustained low-efficiency dialysis, CRRT: continuous renal replacement therapy

	CRRT			SLED			P-value
	N		Mean ± SD of APACHE 2 score	N		Mean ± SD APACHE 2 score	
Non-survivors	27	22.7±7.7		25	25.52±9.34		0.12
Survivors	8	18.12±9.24		7	8.71±2.36		0.15
P-value		0.08			0.19		
Mean comparison of SOFA score among survivors and non-survivors							
	N	Mean+/- SD of SOFA score		N	Mean +/- SD SOFA score		P-value
Non-survivors	27	11.44+/-2.34		25	11.84+/-2.28		0.27
Survivors	8	9.37+/-2.19		7	8.71+/-2.36		0.5
P-value		0.01			0.001		

Among the 35 patients in the CRRT group, eight (22.8%) patients survived, and 27 (77.1%) patients died. The mean SOFA scores of the non-survivors and survivors in the CRRT group were 11.44 ± 2.34 and 9.37 ± 2.19, respectively. The SOFA score among the non-survivors was much greater than that among survivors, and the difference was statistically significant. Among the 32 patients in the SLED group, seven patients survived and 25 died. The mean SOFA scores of the non-survivors and survivors were 11.84 ± 2.28 and 8.71 ± 2.36, respectively. The SOFA score among the survivors was significantly lower than that among the non-survivors (Table 7).

There were significant statistical differences noted between the survivors and non-survivors in both groups with respect to Delta VI and Delta VD (Table 8). Also, there is statistical significance noted between survivors between the CRRT and SLED groups for Delta VI. This table signifies lower Delta VI and Delta VD scores at the initiation of RRT, whether CRRT or SLED, show a favourable prognosis. 

**Table 8:** Mean comparison of Delta VI and Delta VD between survivors and non-survivors Delta VD: difference of vasopressure dependence, Delta VI: difference of vasopressure index, CRRT: continuous renal replacement therapy, SLED: sustained low-efficiency dialysis

	CRRT	Mean ± SD of Delta VI	SLED	Mean ± SD of Delta VI	P-value
Non-survivors	27	42.57 ± 26.1	25	41.34 ± 27.34	0.37
Survivors	8	27.52 ± 10.31	7	15.85 ± 11.14	0.02
P-value		0.04		0.001	
	CRRT	Mean ± SD of Delta VD	SLED	Mean ± SD of Delta VD	P value
Non-survivor	27	73.88 ± 50.60	25	69.72 ± 48.52	0.25
Survivor	8	40.44 ± 16.74		25.04 ± 17.69	0.92
P-value		0.01		0.004	

As the patients were not randomised, a logistic regression was carried out between the survivors and non-survivors in the CRRT and SLED groups for ΔVI and ΔVD to reduce the chance of selection bias. There was no statistically significant difference noted between the two groups except in the SOFA score and ultrafiltration (Table 9).

**Table 9:** Outcome: survivors with non-survivors (logistic regression) SE: standard error, SIG: p-value, Exp(B): odds ratio, PT/INR: prothrombin time/international normalised ratio, APTT: activated partial thromboplastin time, SOFA: Sequential Organ Failure Assessment, APACHE: Acute Physiological Assessment and Chronic Health Evaluation

	B	SE	Sig	Exp(B) odds ratio
Age-group (years)	0.262	1.125	0.816	1.300
Sex	-4.133	2.285	0.70	0.016
Comorbidity	-2.710	1.406	0.054	0.067
Platelet x10^9^/L	0.000	0.014	0.968	1.000
PT/INR	-1.645	1.761	0.350	0.193
APTT (seconds)	-0.124	0.148	0.404	.884
Procalcitonin (ng/ml)	0.003	0.017	0.873	1.003
Lactate (mmol/l)	0.390	0.298	0.191	1.477
Creatinine (mg%)	0.990	0.525	0.059	2.692
pH	-10.147	13.841	0.464	0.000
HCO3 (meq/l)	0.509	0.401	0.205	1.663
Potassium (meq/l)	-1.284	0.819	0.117	0.277
SOFA Score-Predialysis	-2.214	0.786	0.005	.109
APACHE-2 Score-Predialysis	0.378	0.237	0.111	1.459
UF (litre)	0.001	0.001	0.031	1.001
Group	-1.976	2.306	.392	.139
Constant	94.237	100.639	.349	8.446E+40

However, between the CRRT and SLED groups, APACHE-2 score, bicarbonate, pH, procalcitonin, platelet, and comorbidity showed statistical significance (Table 10).

**Table 10:** - Logistic regression analysis comparing CRRT VS SLED SE -Standard Error , Sig- p value , Exp(B) - Odds Ratio

	B	SE	Sig (p)	Exp(B) Odds Ratio
Age group(years)	0.321	0.519	0.536	1.379
sex	0.381	1.010	0.706	1.464
comorbidity	-1.176	0.591	0.047	0.308
Platelet x10^9^/L	0.025	0.011	0.019	1.025
PT/INR	-0.540	1.106	0.626	0.583
APTT(seconds)	-0.075	0.117	0.525	0.928
Procalcitonin(ng/ml)	-0.016	0.014	0.014	0.954
Lactate(mmol/l)	0.367	0.150	0.757	1.443
Creatinine(mg%)	0.073	0.237	0.002	1.076
HCO3(meq/l)	-0.435	0.211	0.039	0.647
Potassium(meq/l)	0.394	0.461	0.392	1.483
SOFA Score-Prdialyis	-0.186	0.329	0.572	0.830
Apache-2 Score-PreDialysis	0.235	0.110	0.034	1.264
UF	0.000	0.000	0.559	1.000
Outcome	-1.456	1.540	0.344	0.233
Constant	-246.690	78.965	0.002	0.000

Filter clotting (18.75% vs. 46.87%, SLED vs. CRRT, respectively) was the most common complication in the two study groups, followed by hypokalemia (15.63% vs. 34.37%), hypophosphatemia, and arrhythmia. The complications appeared to be less common in the SLED group, but the difference was not statistically significant, except for filter clotting (Table 11).

**Table 11:** Comparison of complications between the study groups SLED: sustained low-efficiency dialysis, CRRT: continuous renal replacement therapy

Variables	SLED	CRRT	Chi-square	P-value
Filter clotting	6 (18.75%)	15 (46.87%)	4.514	0.032
Hypokalemia	5 (15.63%)	11 (34.37%)	2.297	0.219
Hypophosphatemia	2 (6.25%)	4 (12.5%)	0.550	0.75
Arrhythmias	3 (9.37%)	6 (18.75%)	0.867	0.35

## Discussion

Among critically ill patients, AKI requiring RRT continues to be associated with very high mortality, ranging from 50% to 80%, despite considerable technological advancements in RRT [4,17,18]. Both CRRT and SLED are used interchangeably; however, they are frequently driven by factors such as physicians’ prerogative, cost, and utilisation of resources, which are primary drivers in developing nations [2].

Currently, there are no guidelines or consensus regarding a preferred RRT modality in a hemodynamically contingent setting. Both these techniques have their own merits and demerits. In comparison to IHD, CRRT is associated with improved hemodynamic stability, greater chances of recovery, better survival, control of volume overload, and azotemia. However, all these studies have compared CRRT to extensive historical control [3]. The disadvantages of CRRT include the need for anticoagulation, proprietary premanufactured solutions, high cost, the need for interruption to allow for off-unit testing, and the need to frequently interrupt the therapy to allow for tests and procedures [2].

SLED is a hybrid technique with features of both CRRT and IHD that is used increasingly in developing countries for the management of AKI with hemodynamic instability. Owing to its easy availability, low cost, and reasonable expertise required in performing this therapy, it is gaining popularity as a preferred modality in most ICUs [13]. Therefore, this study assessed the suitability of SLED as an alternative to CRRT for hemodynamically unstable critically ill ICU patients.

Our study (Table 1) included mainly elderly patients with a median age of 56.17 ± 14.13 years and 59.72 ± 14.08 years in the CRRT and SLED groups, respectively. This was similar to the study by Kitchlu A et al., who reported that the mean age of patients was estimated to be 62 ± 15.3 years and 60.6 ± 17.3 years in the CRRT and SLED groups, respectively [15]. Liaño et al. and Stevens et al. observed similar baseline characteristics in their study [2,19]. AKI is emerging as the leading risk factor in elderly individuals, mainly owing to ageing kidneys along with a cumulative increase in the incidence of comorbidities and medication use, resulting in altered renal hemodynamics. In our study, more males suffered from AKI requiring RRT than females, i.e., 77% and 23% in the CRRT group against 53% and 47% in the SLED group, respectively. Major comorbidities, such as diabetes, hypertension, coronary artery disease, and chronic obstructive pulmonary disease, showed similar frequency distributions in the two groups. Moreover, approximately 73.09% of our cohort had at least one comorbidity at presentation, and the remaining 26.91% had no comorbidity. When we indexed the degree of comorbidities in our study, almost 16.41% had three comorbidities and 20.89% had two. Ali et al. reported a similar pattern of frequency distribution in their study group, i.e., 80%, 24%, and 18% of their cohort had one, two, and three comorbidities, respectively [20].

At baseline, there were no discrepancies between the two groups of the study population (Table 1) with respect to the aetiology of AKI, SOFA score, or laboratory parameters, except total platelet count, which exceeded the level of significance; this signifies similar patient characteristics in both groups, possibly reducing the selection bias. 

We followed the standard criteria for initiating RRT, which included uraemia, severe metabolic acidosis refractory to medical management, oliguria, fluid overload, and hyperkalaemia (Table 2). Our findings are in accordance with those by Ponce et al., who reported that the main reasons for the initiation of RRT were oliguria/fluid overload, uraemia, metabolic acidosis, and hyperkalaemia, in descending order [21]. Mishra SB et al. reported metabolic acidosis as the major driver for the initiation of RRT, followed by hyperkalaemia and fluid overload [12].

Hemodynamic tolerance is a major concern in critically ill patients with AKI. The traditional usage of MAP as a tool for establishing hemodynamic tolerability has been questioned by various authors. A landmark study by Asfar et al. led to the foundation for titration of vasopressors according to MAP, which was later incorporated into the Surviving Sepsis Guidelines [22]. In their study, the patients were grouped as those with a higher MAP of more than 85 mm of Hg and those with a lower MAP of 70 to 84 mm of Hg. Their study did not find any significant difference between the two groups in terms of hard outcomes such as 28-day and 90-day mortalities. Moreover, the study revealed a progressive increase in mortality with an increase in the duration of exposure to a lower MAP of <65 mmHg [22].

The objective and comparable concept of the VI and VD for hemodynamic instability assessment was proposed by Mishra SB et al. [12-13]. The advantage of these two parameters nullifies the effect of the measurement of MAP alone in hemodynamically unstable patients with AKI. They proposed that patient-to-patient variability does exist and that patients with chronic hypertension and diffuse atherosclerosis require a higher MAP for better organ perfusion; hence, a higher MAP was targeted. Delta VI ΔVI and ΔVD are more effective parameters, as they include the dose of inotropic requirements, which is better for titrating the dose of inotropes. 

Kielstein et al. and Baldwin et al. compared CRRT and SLED in critically ill patients for hemodynamic stability and concluded that both are comparable in the correction of acidosis at the end of RRT [23,24]. In our study, hemodynamics in terms of VI, VD, MAP, Delta VI, and Delta VD were similar in both groups and did not significantly differ (Table 3). A metanalysis published by Rabindranath et al. revealed that compared to patients receiving SLED, those receiving CRRT were less likely to need escalation of their inotropic support, maintaining a significantly greater MAP [25]. However, our analysis revealed discordant observations, with similar inotropic support escalation in both groups. A comparison of the parameters by logistic regression analysis and ANOVA revealed no statistically significant difference (Tables 4, 5).

Early clinical deterioration, defined as death within 48 hours, was observed in 45.71% and 50% of patients in the CRRT and SLED cohorts, respectively, in our study (Table 6). The results obtained in our study were congruent with those reported by Kitchlu et al., with 45.2% in the CRRT group and 39.2% in the SLED group [15]. The SOFA score deteriorated in non-survivors at 48 hours from 12.55 ± 2.42 in the CRRT cohort and 12.77 ± 2.22 in the SLED cohort to 13.72 ± 1.79 and 14 ± 2.22, respectively (Table 7). The VI, VD, ∆VI, ∆VD, and SOFA scores were significantly higher in non-survivors than in survivors at the time of initiation of RRT. The most likely explanation for this finding is that patients with higher pre-initiation vasopressor requirements showed greater hemodynamic worsening during SLED. Since VD ≥25 showed a good sensitivity, albeit only with modest specificity, it may be used as a predictor of a hemodynamically intolerable SLED session. Similarly, Delta VD was lower in survivors (<40) and >40 in non-survivors in our cohort (Table 8). However, owing to the small sample in this study, a relevant relationship could not be established.

Finally, in another trial by Schoenfelder et al., it was observed that initial CRRT was associated with increased rates of renal recovery. CRRT does not significantly differ from intermittent renal replacement therapy in terms of change in MAP, hypotensive episodes, hemodynamic instability, and length of stay [26]. 

AKI poses a very heavy burden on morbidity and mortality. In our study, out of 67 patients who underwent RRT, the total mortality rate at 28 days was 77.14 % in the CRRT cohort and 78.12% in the SLED cohort. 

Ponce et al. reported a mortality rate of 71.9% among patients undergoing CRRT, while an Indian study by George et al. reported a mortality rate of 84% in the continuous venovenous hemodiafiltration (CVVHDF) group compared to 72% in the peritoneal dialysis (PD) group [21,27]. Kitchlu et al. reported all-cause mortality rates of 54% and 61% in the SLED and CRRT groups, respectively, at 30 days [15]. Schwenger V et al., in their single-centre study, compared PIRRT to CVVH in 232 critically ill patients. There was no difference in 90-day all-cause mortality between the PIRRT group and the CVVH group. Patients treated with PIRRT required fewer days of mechanical ventilation, required fewer days in the ICU, and received fewer blood transfusions, resulting in an overall lower cost of therapy [28]. A subsequent meta-analysis of seven randomised, controlled trials and 10 observational studies comparing PIRRT with CRRT revealed no difference in mortality or recovery of kidney function associated with the modality of therapy [29].

Long-term RRT dependence did not differ between the two groups in seven randomised trials [30]. In our study, the frequency of RRT dependence at 28 days was 40% and 42.85% in the CRRT and SLED cohorts, respectively. Similarly, Kitchlu et al. reported that RRT dependence at 30 days was 32.8% and 38.2% in the CRRT and SLED groups, respectively [15]. Ponce et al. reported that 22.5% of patients presented with renal function recovery, and 5.6% of patients remained on dialysis after 30 days [21].

Recovery of kidney function appears to be the same with CRRT and IHD. Although some studies report better recovery with CRRT, these reports only evaluated renal recovery in patients who survived, thereby failing to account for mortality differences between groups. When the analysis combined mortality and non-recovery of renal function, both groups showed similar recovery of function. These observations were confirmed in a meta-analysis by Scheinder et al. that included 3971 survivors of RRT-requiring AKI. A pooled analysis of 16 observational studies (n = 3499) revealed a higher rate of dialysis dependence associated with IHD, but analysis of seven randomised trials showed no difference in the recovery of kidney function between groups [30]. To optimise the relevance of statistical parameters in a non-randomised trial, we performed logistic regression analysis for survivors and non-survivors as well as between the two RRT modalities, i.e., CRRT vs SLED (Table 9). The analysis revealed that lactate level, serum creatinine at baseline, serum bicarbonate at baseline, and APACHE-2 score showed an odds ratio of more than one. Similarly, when we compared the odds of parameters comparing CRRT with SLED, age, sex, lactate level at baseline, serum pH at baseline, serum Potassium, and APACHE-2 score showed values more than one (Table 10).

Hypotension, abnormalities in serum electrolytes, albumin, calcium, and phosphate, hypothermia, infection and bleeding, sub-therapeutic antibiotic concentration, arrhythmia, membrane bio incompatibility, and complications due to vascular access are well-recognised complications of RRT. We investigated the most common parameters, namely, filter clotting, hypokalaemia, hypophosphatemia, and arrhythmia. Filter clotting was the most frequent complication observed in our study. It was more common in the CRRT group than in the SLED group; it was observed in 46.87% of the patients in the CRRT group and 18.75% of those in the SLED group (Table 11). Hypokalaemia and hypophosphatemia were observed in 34.37% and 12.5% of the patients in the CRRT group, respectively, while they were observed in 15.63% and 6.25% in the SLED group, respectively. The incidence of arrhythmia was 18.75% and 9.37% between the CRRT and SLED groups, respectively. 

This study had several limitations. First, this was a single-centre observational study comprising a relatively small number of patients with possible allocation bias. A randomised control trial with a larger sample size would have been desirable. Second, we did not compare outcome parameters such as length of ICU stay, ventilation duration, and recovery of renal function beyond one month. The strength of the present study lies in the objective assessment of hemodynamic parameters; however, comparison of modalities in terms of efficacy, fluid balance in the interdialytic period and equivalent renal urea clearance, and cost analysis should have been carried out, which was not done in the current study. In addition, a single-centre setup and small sample size weakened the ability to detect a link between mortality and renal function recovery.

Nevertheless, despite these shortcomings, we have attempted to objectively define the hemodynamic tolerability and efficacy of SLED in patients with AKI and septic shock better than the relatively more costly modality, i.e., CRRT.

## Conclusions

Titration of MAP alone was not sufficient to define hemodynamic instability in critically ill patients in the ICU. Hence, the current study reemphasised that the vasopressor index and vasopressor dependency are better indices to assess the hemodynamic stability in critically ill patients with AKI requiring RRT. Both SLED and CRRT showed similar hemodynamic tolerability, mortality profile, and short-term renal recovery in patients with AKI requiring RRT in our study. SLED showed a lower incidence of filter clotting, hypokalemia, and hypophosphatemia when compared with CRRT, although without a statistically significant difference. 
